# Electrocardiographic Characteristics and Ablation Outcomes Associated With Para-Hisian Ventricular Arrhythmias

**DOI:** 10.1016/j.jacasi.2024.11.012

**Published:** 2025-01-14

**Authors:** Anugrah Nair, Jenish P. Shroff, Lukah Q. Tuan, Adriana Tokich, Deep Chandh Raja, Abhinav Mehta, Walter P. Abhayaratna, Prashanthan Sanders, Francis E. Marchlinski, Kalyanam Shivkumar, Rajeev K. Pathak

**Affiliations:** a Australian National University, Canberra, Australian Capital Territory, Australia; b Canberra Heart Rhythm Centre, Canberra, Australian Capital Territory, Australia; c Centre for Heart Rhythm Disorders, University of Adelaide and Royal Adelaide Hospital, Adelaide, Australia; d Cardiovascular Division, Electrophysiology Section, Hospital of the University of Pennsylvania, Philadelphia, Pennsylvania, USA; e UCLA Cardiac Arrhythmia Center and EP Programs, UCLA Health System, Los Angeles, California, USA

**Keywords:** catheter ablation, electro-anatomical mapping, myocardial scar, Para-Hisian, right ventricular outflow tract ventricular, ventricular arrhythmias

## Abstract

**Background:**

Ventricular arrhythmias (VAs) near the His-bundle comprise 9% of unexplained VAs and present challenges for ablation caused by the risk of atrioventricular block.

**Objectives:**

The authors studied the electrocardiographic (ECG) and electrophysiological characteristics of Para-Hisian (PH) VAs, comparing them with septal right ventricular outflow tract VAs.

**Methods:**

From 210 patients with VAs between 2018 and 2024, 31 (14.7%) with PH-VAs and 23 (10.9%) with septal right ventricular outflow tract VAs were included. ECG characteristics of both were compared, and features differentiating left and right PH and supra- and infra-Hisian VAs were identified.

**Results:**

Of 31 patients, 15 had VAs from the right PH site and 16 from the left. Median follow-up was 15 months (Q1-Q3: 14-21 months) for left infra-Hisian, 16 months (Q1-Q3: 14-20 months) for left supra-Hisian, and 14 months (Q1-Q3: 14-16 months) for right infra-Hisian and right supra-Hisian VAs (Q1-Q3: 14-15 months). PH-VAs had narrower QRS complexes (134 ± 19.6 ms vs 169 ± 24 ms; *P* < 0.05), R-wave in lead aVL (100% [31 of 31] vs 4.3% [1 of 23]; *P* < 0.001), and earlier R-wave transition at or before lead V_3_ (80.6% [25 of 31] vs 47.8% [11 of 23]; *P* < 0.05). Left PH-VAs had earlier R-wave transition at lead V_2_ (50% [8 of 16] vs 20% [3 of 15]; *P =* 0.036). Right PH VAs had deeper S-wave relative to the preceding sinus beat in lead V_1_ (73.3% [11 of 15] vs 37.5% [6 of 16]; *P =* 0.04) and lead aVR (80% [12 of 15] vs 56.3% [9 of 16]; *P =* 0.01). Postprocedure heart block occurred in 1 patient.

**Conclusions:**

PH-VAs exhibit unique ECG features based on their origins, and can be effectively treated without affecting atrioventricular conduction.

Premature ventricular contractions (PVCs) and ventricular arrhythmias (VAs) along with potentially debilitating symptoms can lead to heart failure.^1^ Over the last 20 years catheter ablation (CA) has advanced significantly. VAs near the His-bundle area account for around 9% of VAs. Treating these arrhythmias with CA is challenging caused by the risk of atrioventricular (AV) block, necessitating a systematic approach to mapping and ablation.^2^

The His bundle penetrates the membranous septum through the central fibrous body. The Para-Hisian (PH) region is considered the area of the right ventricle (RV) or left ventricle (LV) septum where the site of origin (SOO) is recorded near the His potential or within 10 mm of the His cloud created during electroanatomical mapping.^3^ These are termed supra-Hisian and infra-Hisian based on their superior or inferior SOO in relation to the His cloud. Technological innovations, including 3-dimensional (3D) mapping and periprocedural imaging, have greatly improved our understanding of SOO of these VAs and the anatomical proximity of adjacent structures that could be used for CA.^4^

Because of anatomical proximity, the electrocardiographic (ECG) and electrophysiological (EP) features of PH-VAs are similar to those from the lower septal right ventricular outflow tract (RVOT), the coronary cusp region and LV septum below the aortic valve.^5^, ^6^, ^7^, ^8^ This paper systematically examines ECG and EP features of PH-VAs and compares them with septal RVOT VAs. Additionally, we subdivided these VAs into left and right PH VAs, as well as supra- and infra-Hisian VAs. Moreover, we looked at the ablation outcomes in terms of improvement of LV function, reducing VA burden, and enhancement of the patients’ quality of life (QOL).

## Methods

### Study population

This is a single-centre observational study. A prospective database encompassing 210 patients who underwent VA ablation at our institution between 2018 and 2024 were analyzed. Among these, 31 cases (14.7%) were identified as originating within 10 mm of the His potential on mapping and were included. Additionally, 23 patients with VAs arising from the septal RVOT were also included. Patients with VAs originating from other areas of LV and RV were excluded.

### 12-lead ECG analysis

During spontaneous or induced VAs, 12-lead ECGs were recorded at paper speeds of 25 mm/s, with inferior leads placed over arms and legs and positions of leads were consistent during acquisition of electrograms using the BARD LABSYSTEM PRO EP Recording System (Boston Scientific Corporation). The ECGs were analyzed blindly by 2 independent investigators and cross-checked by a third (A.N., R.P., J.S.), utilizing the E-scribe ECG analysis system (Hill-Rom Holdings, Inc) and an electronic caliper for accuracy.

The analysis focused on the duration of the QRS complex and the preceding sinus beat in inferior leads, evaluating patterns of left bundle branch block (QS, rS) or right bundle branch block (RBBB) (rsR, qR) in lead V_1_. It assessed the presence, magnitude, and ratio of R and S waves, the R/S ratio, QRS notching in all leads, and inferior lead discordance (positive QRS complex in lead II and negative QRS complex in III or vice versa). VAs from the PH region were compared with septal RVOT VAs, and features distinguishing left vs right PH and supra- or infra-Hisian VAs were identified.

### Electrophysiologic testing, mapping, and ablation

Electroanatomic and activation mapping were used to define the PH region and catheter position. EP testing was performed in a fasting, nonsedated state. Bipolar electrograms (EGMs) were recorded with a 3.5-mm tip mapping/ablation catheter (Smart touch surround flow [STSF] or Q DOT MICRO Biosense Webster [BW]) with a 30 to 500 Hz bandpass filter, and unipolar EGMs with 0.5 to 500 Hz bandpass filter. A 10-F intracardiac echocardiography (ICE) probe (SOUNDSTAR, Johnson and Johnson Medical NV/SA) was used for direct catheter visualization and Carto sound map was created for all patients. The basal septum and its relationship to the coronary cusp were identified. Spontaneous PVCs were monitored, and isoprenaline was used if no VAs were initially present.

3D mapping with contemporary versions of CARTO 3 System (Biosense Webster, Inc) was used for activation and pace mapping. Radiofrequency (RF) ablation was performed with an 8-F quadripolar catheter (STSF/Q DOT MICRO) with a deflectable bidirectional (D/F) tip and a 3.5-mm distal electrode. The optimal ablation site was determined by the earliest endocardial activation and best pace mapping, requiring a match of 11 of 12 criteria. If the site was near the His-bundle, RF energy was cautiously delivered, starting with low power and gradually increasing, at least 5 mm from the largest His-bundle potential.

If VA suppression or acceleration occurred in the first 10 seconds, RF delivery continued for an additional 30 to 60 seconds. If no effect was noted after 10 to 15 seconds, RF delivery was stopped, and the catheter repositioned. RF was immediately halted if AH prolongation, fast junctional rhythms, RBBB, or transient heart block occurred. The anatomic position of the catheter tip was verified using ICE imaging, electroanatomic mapping (EAM), computed tomography integration, and fluoroscopy. The SOO was classified as right supra- or infra-Hisian and left supra or infra-Hisian based on the successful ablation site.

### Myocardial function and scar assessment

All patients underwent baseline and 1-year transthoracic echocardiography (Vivid E95, GE Healthcare) to assess ejection fraction (EF). Presence of myocardial scar was assessed using cardiac magnetic resonance imaging (1.5-T Siemens Sola wide-bore scanner) integrated with circle CVI imaging software (Circle Cardiovascular Imaging Inc). Additionally, ICE and EAM (low-voltage signals around the area of earliest activation) were used to identify scar during the procedure.

### QOL assessment

QOL improvements at baseline and 1 year were evaluated using the Short Form health survey (SF) 36 and EuroQol group (EQ-5D 5L) questionnaires, which looked into multiple health dimensions, including general health status, mobility, self-care, daily activities, pain/discomfort, and anxiety/depression.

### Outcomes

Treatment outcomes were measured as follows:

1. Single procedure drug-free outcomes: Freedom from arrhythmia without antiarrhythmic drugs (AADs) post–index ablation at 3-month follow-up.
2. Multiple procedures drug-free outcomes: Freedom from arrhythmia without AAD after >1 ablation.
3. Total outcome (single or multiple) with medications: Freedom from arrhythmia with AAD and ≥1 ablation procedure. Procedural success was defined as freedom from arrhythmia 72 hours postablation without AAD, and a reduction in target VAs by 90% (compared with baseline) or an absolute decrease of <5% without AAD for at least 3 months.

### Follow-up

All patients underwent 48-hour telemetry immediately postprocedure and were monitored for at least 2 years with echocardiography and 72-hour Holter for PVC burden at 3 and 12 months during the first year, then annually. Data were standardized, encoded, anonymized, and stored in a prospectively maintained VA database. The study adheres to the Declaration of Helsinki and was approved by the ACT Health Human Research Ethics Committee.

### Statistical analysis

Data are presented as mean ± SD or median (Q1-Q3) as appropriate. Continuous variables were analyzed using paired Student's *t*-tests, and categorical variables were assessed with the chi-square test. The agreement between ECG measurements from 2 blinded observers was evaluated using the intraclass correlation coefficient (2-way random model) and an F-test to determine if the correlation was >0. Kappa statistics were used to measure classification agreement, with an acceptable variability of ±5 ms in timing and ±0.1 mV in voltage. Statistical significance was defined as *P <* 0.05, and analyses were performed using IBM SPSS version 23.0.

## Results

### Baseline characteristics

Among 210 patients undergoing VA ablation, 31 (22 men, mean age 59 ± 7.7 years) had VAs from the PH region. Table 1 details the baseline characteristics of left supra-Hisian, left infra-Hisian, right supra-Hisian, and right infra-Hisian VAs. Common symptoms included palpitations (n = 31), dizziness (n = 29), and syncope (n = 3). Medications included beta-blockers (n = 21), AADs (n = 10), and other heart failure medications (n = 13). Myocardial scar was detected in 16 patients (ICE: 8, CMR: 14, EAM: 11). VAs originated from the left PH region in 16 patients (supra-Hisian: 6; infra-Hisian: 10) and the right PH region in 15 patients (supra-Hisian: 7; infra-Hisian: 8).

**Table 1:** Baseline Characteristics of Para-Hisian Ventricular Arrhythmias

	Left Supra-Hisian	Left Infra-Hisian	*P* Value	Right Supra-Hisian	Right Infra-Hisian	*P* Value
Male	4 (66.7)	6 (60)	0.795	5 (71.4)	7 (87.5)	0.452
Female	2 (33.3)	4 (40)	0.795	2 (28.6)	1 (12.5)	0.452
Mean age, y	61 ± 9.6	56.4 ± 9.5	0.366	58 ± 5.1	61 ± 5.4	0.293
Hypertension	4 (66.7)	6 (60)	0.667	4 (57.1)	2 (25)	0.207
Type 2 diabetes	1 (16.7)	1 (10)	0.398	1 (14.3)	1 (12.5)	0.188
Dyslipidemia	2 (33.3)	4 (40)	1.000	4 (57.1)	4 (50)	0.725
Chronic kidney disease	1 (16.7)	1 (10)	0.205	2 (28.6)	1 (12.5)	0.252
Coronary artery disease	3 (50)	3 (30)	0.264	4 (57.1)	3 (37.5)	0.429
Mixed cardiomyopathy	2 (33.3)	1 (10)	0.063	2 (28.6)	1 (12.5)	0.224
Dilated cardiomyopathy	4 (66.7)	2 (20)	0.205	2 (28.6)	2 (25)	0.928
Mean BMI, kg/m^2^	30 ± 5.7	30.8 ± 8.1	0.835	29.6 ± 3.3	27 ± 4.2	0.210
Presence of myocardial scar	3 (50)	4 (40)	0.705	5 (71.4)	4 (50)	0.414

Values are n (%) or mean ± SD.

BMI = body mass index.

### Comparison of Para-Hisian VAs vs septal RVOT VAs

**Figure 1:**
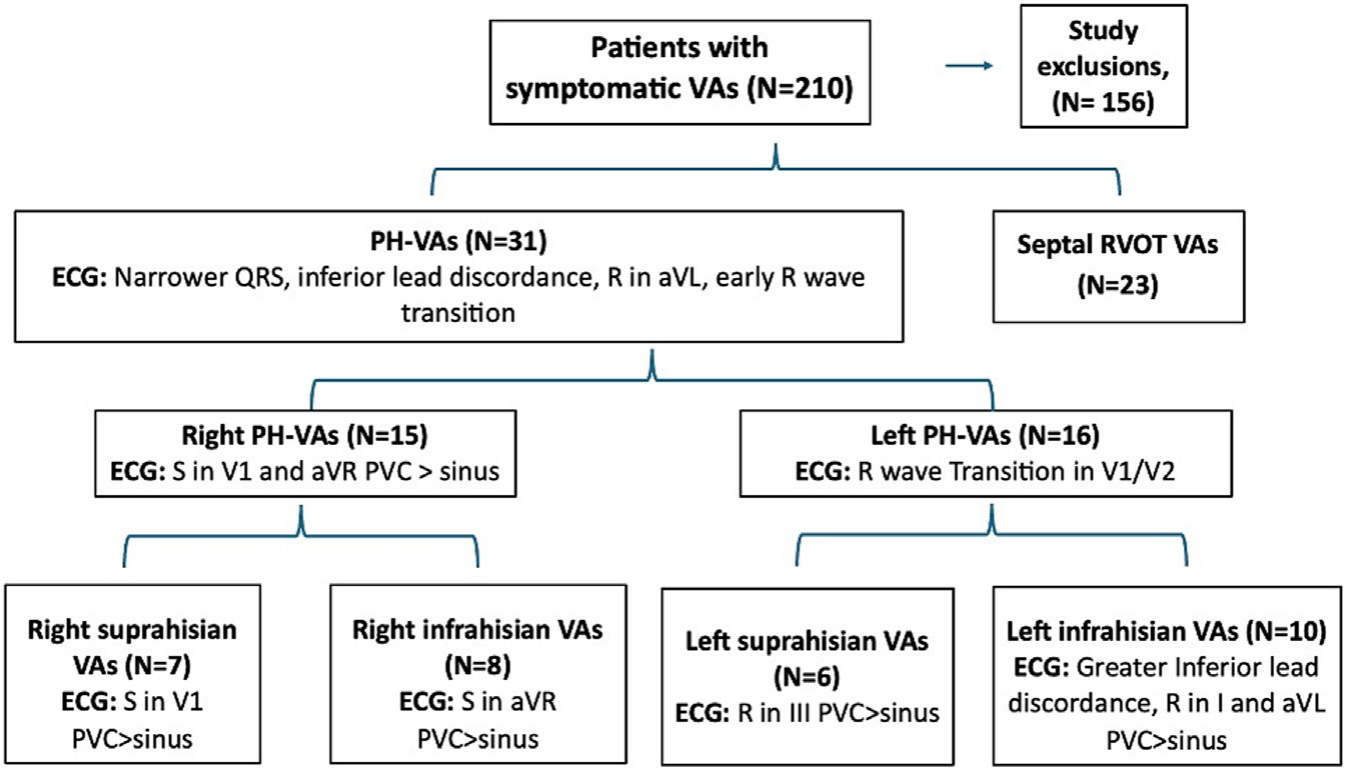
Flow Diagram of Patient Recruitment This figure outlines the selection process of patients with symptomatic ventricular arrhythmias (VAs) and their categorization into subgroups. Of the 210 patients, 31 were identified with Para-Hisian (PH)-VAs, and 23 with septal right ventricular outflow tract (RVOT) VAs. Among the PH-VA patients, 15 had right-sided PH VAs and 16 had left-sided PH VAs. PH-VAs demonstrated narrower QRS complexes (134 ± 19.6 ms vs 169 ± 24 ms; *P* < 0.05), 100% of PH-VAs had an R-wave in lead aVL compared with 4.3% of RVOT-VAs (*P <* 0.001), and PH-VAs exhibited an earlier R-wave precordial transition at or before lead V_3_ (80.6% vs 47.8%; *P* < 0.05). PVC = premature ventricular contraction; RBBB = right bundle branch block.

**Figure 2:**
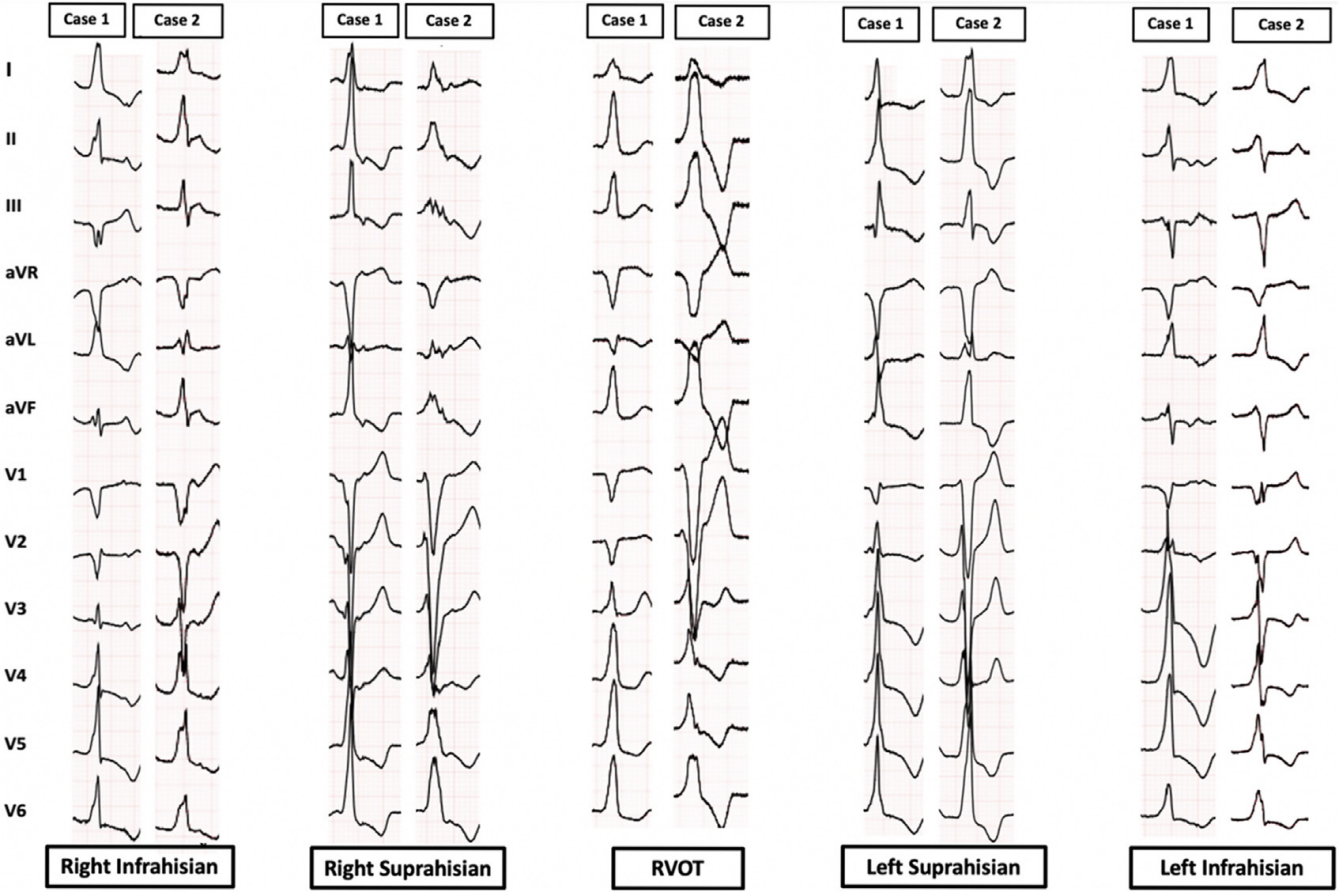
The Electrocardiographic Characteristics of Para-Hisian Ventricular Arrhythmias and Septal RVOT Ventricular Arrhythmias The figure highlights the comparison of the distinct electrocardiogram characteristics of Para-Hisian ventricular arrhythmias and septal right ventricular outflow tract (RVOT) ventricular arrhythmias, Para-Hisian ventricular arrhythmias displayed narrower QRS complexes and more frequent inferior lead discordance. In addition, Para-Hisian ventricular arrhythmias consistently exhibited R waves in lead aVL. A QS pattern in lead V_1_ was significantly less common in Para-Hisian ventricular arrhythmias compared with septal RVOT ventricular arrhythmias.

Figures 1 and 2 highlights electrocardiographic differences and Table 2 presents comparative analysis between PH-VAs (n = 31) and septal RVOT VAs (n = 23). PH-VAs exhibited narrower QRS complexes (133 ± 18.6 ms vs 162.5 ± 11 ms; *P* < 0.001), more frequent inferior lead discordance (67.7% [21 of 31] vs 0% [0 of 23]; *P* < 0.001), and consistent R-wave presence in lead aVL (100% [31 of 31] vs 4.3% [1 of 23]; *P* < 0.001). PH-VAs also showed earlier R-wave precordial transition characterized by transition before the preceding sinus beat (90.3% [28 of 31] vs 39.1% [9 of 23]; *P* < 0.001) and greater R-wave amplitude in lead I (83.9% [26 of 31] vs 17.4% [4 of 23]; *P* < 0.001), lead aVL (74.2% [23 of 31] vs 4.3% [1 of 23]; *P* < 0.001) and lead V_2_ (64.5% [20 of 31] vs 17.4% [4 of 23]; *P* < 0.001). A QS pattern in lead V_1_ was observed in 25.8% (8 of 31) patients with PH-VAs compared with 95.65% (22 of 23) patients with septal RVOT VAs (*P <* 0.001). Additionally, 25.8% (8 of 31) of PH-VA patients had a QRS duration >150 ms. Among the 16 patients with myocardial scar, 62.5% (10 of 16) patients had a QRS duration >150 ms, and all exhibited notching of the QRS complexes on their ECGs. Notching was present in multiple leads in 9 patients (Supplemental Figure 1), whereas 7 patients showed notching in a single lead.

**Table 2:** Electrocardiographic Comparison of Para-Hisian and Septal RVOT Arrhythmias

	RVOT (n = 23)	Para-Hisian (n = 31)	*P* Value
VA QRS duration, ms	162.5 ± 11.1	133.3 ± 18.6	0.001
Preceding sinus beat QRS duration, ms	98 ± 14.9	127 ± 26.7	0.001
QRS duration >150 ms	23 (100.0)	8 (25.8)	0.001
LBBB pattern in V_1_	23 (100.0)	25 (80.6)	0.027
PVC transition before preceding sinus beat	9 (39.1)	28 (90.3)	0.001
PVC transition at or before lead V_3_	11 (47.8)	25 (80.6)	0.012
Inferior lead discordance	0 (0.0)	21 (67.7)	
Presence of R-wave in lead aVL	2 (8.7)	31 (100.0)	0.001
R-wave in lead I PVC > sinus beat	4 (17.4)	26 (83.9)	0.001
R-wave in lead aVL PVC > sinus beat	1 (4.3)	23 (74.2)	0.001
R-wave in lead V_2_ PVC > sinus beat	4 (17.4)	20 (64.5)	0.001

Values are mean ± SD or n (%).

LBBB = left bundle branch block; PVC = premature ventricular contraction; RBBB = right bundle branch block; VA = ventricular arrhythmia.

### Comparison of left vs right para-Hisian VAs

Figure 2 and Table 3 detail the differences between left (n = 16) and right (n = 15) PH-VAs. Left PH-VAs had an earlier R-wave transition in lead V_2_ (50% [8 of 16] vs 20% [3 of 15]; *P =* 0.03) and a larger R-wave in lead V_2_ (81.3% [13 of 16] vs 46.7% [7 of 15]; *P =* 0.04). Left PH-VAs more commonly exhibited RBBB morphology in lead V_1_ (31.3% [5 of 16] vs 0% [0 of 15]; *P =* 0.02). Conversely, right PH-VAs had a larger S-wave in lead V_1_ (73.3% [11 of 15] vs 37.5% [6 of 16]; *P =* 0.04) and lead aVR (80% [12 of 15] vs 56.3% [9 of 16]; *P =* 0.01).

**Table 3:** Electrocardiographic Comparison of Left and Right Para-Hisian Ventricular Arrhythmias

	Left PH-PVC (n = 16)	Right PH-PVC (n = 15)	*P* Value
PVC QRS duration, ms	129.19 ± 18.53	137.7 ± 18.34	0.911
Preceding sinus beat QRS duration, ms	125.8 ± 20.3	128.3 ± 25.2	0.764
PVC R-wave transition at lead V_2_	8 (50.0)	3 (20.0)	0.036
R-wave transition at or after lead V_3_	2 (12.5)	4 (26.7)	0.325
LBBB morphology in V_1_	11 (68.8)	14 (93.3)	0.089
RBBB morphology in V_1_	5 (31.3)	0 (0)	
R-wave in lead V_1_ PVC > sinus beat	7 (43.8)	1 (6.7)	0.020
R-wave in lead V_2_ PVC > sinus beat	13 (81.3)	7 (46.7)	0.047
S-wave in aVR PVC > sinus beat	9 (56.3)	12 (80.0)	0.016
S-wave in lead V_1_ PVC > sinus	6 (37.5)	11 (73.3)	0.049

Values are mean ± SD or n (%).

PH = Para-Hisian; other abbreviations as in Table 2.

### Comparison of right infra-Hisian VAs vs right supra-Hisian VAs

**Figure 3:**
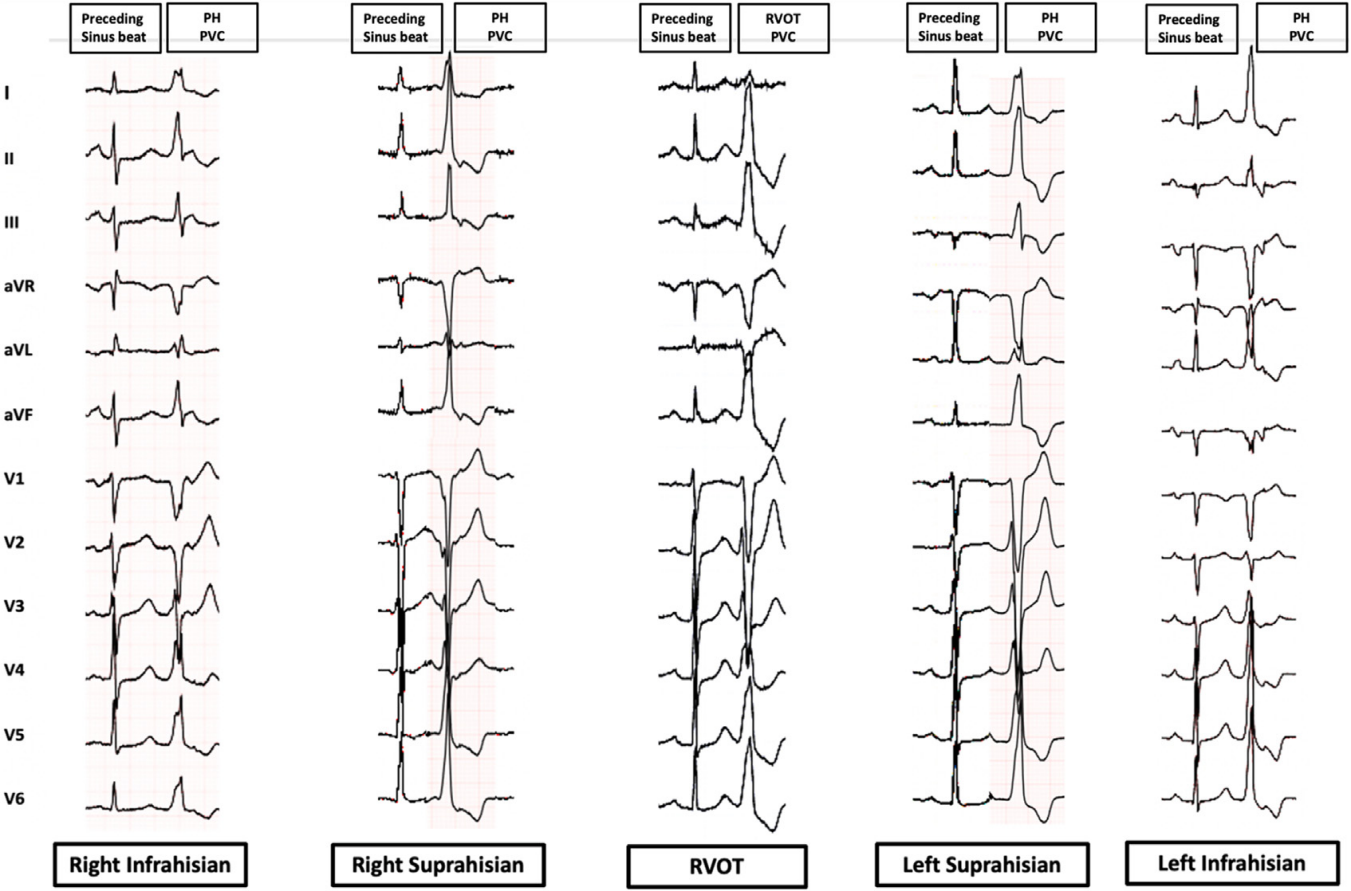
The Electrocardiographic Characteristics of Ventricular Arrhythmias and the Preceding Sinus Beat This figure highlights the electrocardiographic distinctions between Para-Hisian (PH) ventricular arrhythmias and septal right ventricular outflow tract (RVOT) ventricular arrhythmias compared with preceding sinus beat. The first electrocardiogram pattern represents the sinus beat, while the second illustrates the ventricular arrhythmia. Left supra-Hisian ventricular arrhythmias demonstrated a larger R-wave in lead III, while infra-Hisian ventricular arrhythmias showed greater inferior lead discordance and larger R waves in leads I and aVL. Right supra-Hisian PH ventricular arrhythmias showed a larger S-wave in lead V_1_, and infra-Hisian ventricular arrhythmias had a larger S-wave in lead aVR. PVC = premature ventricular contraction.

**Figure 4:**
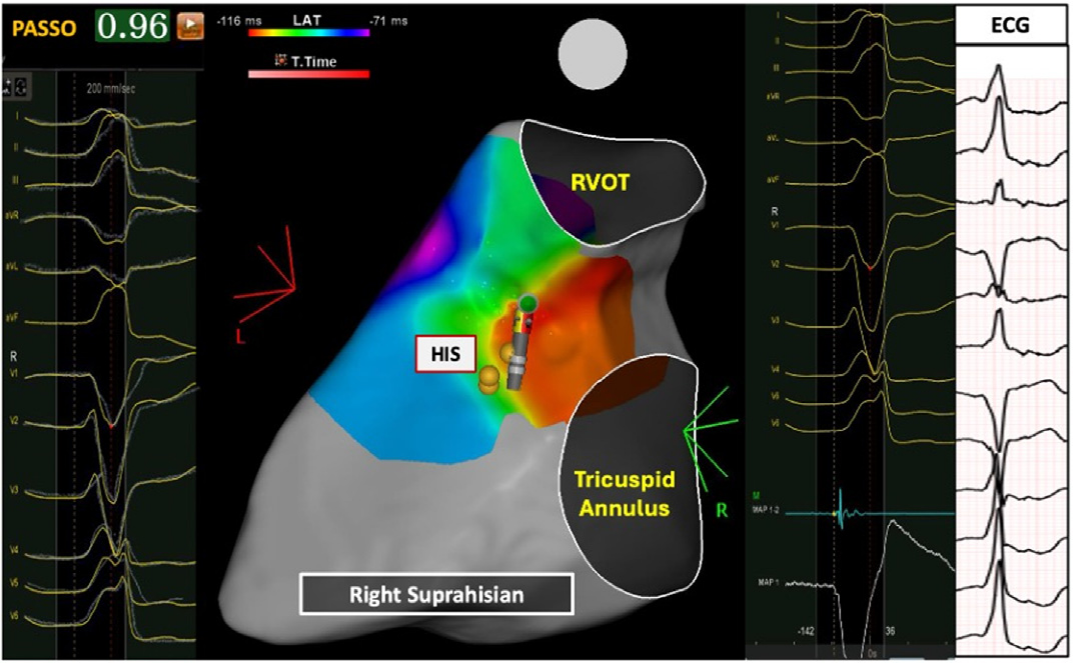
The Electroanatomical Features of Right Supra-Hisian Ventricular Arrhythmias This figure illustrates the electroanatomical location of right supra-Hisian ventricular arrhythmias, highlighting corresponding electrogram, electrocardiogram (ECG), and best pace match. The ECG, recorded at the same speed as the electrogram, shows a 96% pace match at the ablation site, with early signals marked in red. Right supra-Hisian ventricular arrhythmias exhibited a larger S-wave in lead V_1_ compared with the preceding sinus beat (85.7% vs 62.5%; *P =* 0.03). RVOT = right ventricular outflow tract.

Figures 3 and 4 and Table 4 highlight differences between right infra-Hisian (n = 8) and supra-Hisian VAs (n = 7). Right infra-Hisian VAs had a larger S-wave (vs preceding sinus beat) in lead aVR (100% [8 of 8] vs 57.1% [4 of 7]; *P =* 0.03), whereas right supra-Hisian VAs had a larger S-wave (vs preceding sinus beat) (Supplemental Figure 2) in lead V_1_ (85.7% [6 of 7] vs 62.5% [5 of 8]; *P =* 0.03) (Supplemental Figure 3).

**Table 4:** Electrocardiographic Comparison of Different Para-Hisian VAs

	Left Supra-Hisian	Left Infra-Hisian	*P* Value	Right Supra-Hisian	Right Infra-Hisian	*P* Value
VA QRS duration, ms	133.86 ± 20.61	126.39 ± 17.68	0.033	135.47 ± 21.35	139.64 ± 18.34	0.310
Preceding sinus beat QRS duration, ms	131.3 ± 21.1	122.6 ± 21.7	0.440	124.7 ± 26.5	131.3 ± 26.2	0.630
LBB morphology in V_1_	5 (83.3)	6 (60)	0.346	6 (85.7)	8 (100)	0.120
RBBB morphology in V_1_	1 (16.7)	4 (40)	0.050	0 (0)	0 (0)	
R-wave transition at or before lead V_2_	2 (33.3)	6 (60)	0.316	3 (43)	0 (0)	
R-wave transition at lead V_3_	3 (50)	3 (30)	0.438	1 (14.3)	7 (87.5)	0.001
Inferior lead discordance	2 (33.3)	8 (80)	0.006	4 (57.1)	7 (87.5)	0.641
R-wave in lead I PVC > sinus beat	4 (66.6)	10 (100)	0.045	5 (71.4)	7 (87.5)	0.878
R-wave in lead III PVC > sinus beat	5 (83.3%)	3 (30)	0.042	3 (42.9)	4 (50)	0.637
R-wave in lead aVL PVC > sinus beat	3 (50)	9 (90)	0.016	5 (71.4)	7 (87.5)	0.432
R-wave in lead V_1_ PVC > sinus beat	2 (33.3)	5 (50)	0.527	1 (14.3)	0 (0)	
R-wave in lead V_2_ PVC > sinus beat	3 (50)	10 (100)	0.016	5 (71.4)	2 (25)	0.070
S-wave in in aVR PVC > sinus beat	3 (50)	6 (60)	0.705	4 (57.1)	8 (100)	0.030
S-wave in lead V_1_ PVC > sinus beat	3 (50)	3 (30)	0.438	6 (85.7)	5 (62.5)	0.032

Values are mean ± SD or n (%).

Abbreviations as in Table 2.

### Comparison of left infra-Hisian VAs vs and left supra-Hisian VAs

**Figure 5:**
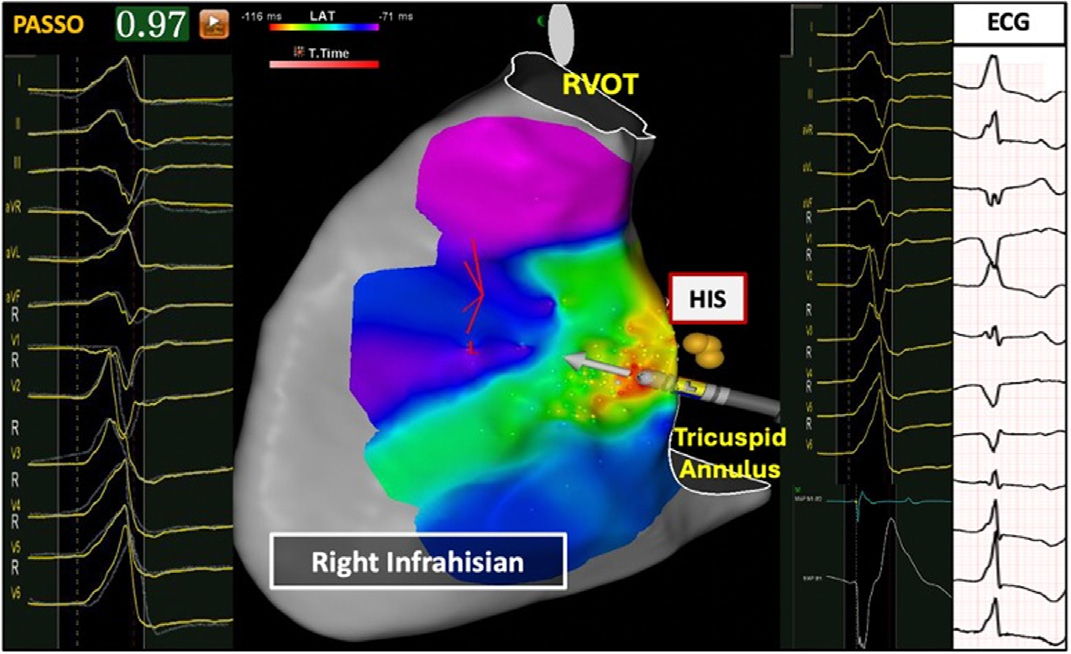
The Electroanatomical Features of Right Infra-Hisian Ventricular Arrhythmias This figure illustrates the electroanatomical location of right infra-Hisian ventricular arrhythmias, showing corresponding electrograms, ECG, and best pace match. The ECG, recorded at the same speed as the electrogram, reveals a 97% pace match at the ablation site, with early signals marked in red. Right infra-Hisian ventricular arrhythmias displayed a larger S-wave in lead aVR compared with the preceding sinus beat (100% vs 57.1%; *P =* 0.03). Abbreviations as in Figure 4.

**Figure 6:**
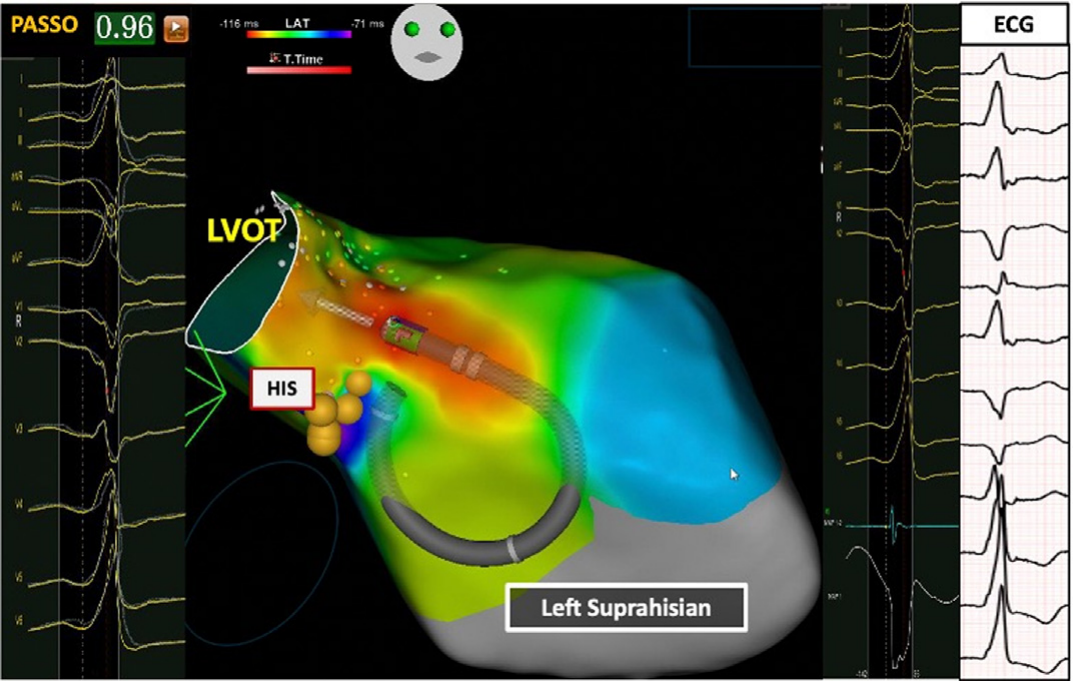
The Electroanatomical Features of Left Supra-Hisian Ventricular Arrhythmias This figure illustrates the electroanatomical location of left supra-Hisian ventricular arrhythmias, showcasing corresponding electrograms, electrocardiogram (ECG), and best pace match. The ECG, recorded at the same speed as the electrogram, reveals a 96% pace match at the ablation site, with early signals marked in red. The catheter orientation was adjusted to accommodate the patient’s breathing. Left supra-Hisian ventricular arrhythmias exhibited a larger R-wave amplitude in lead III compared with the preceding sinus beat (83.3% vs 30%; *P =* 0.042). LVOT = left ventricular outflow tract.

Figures 5 and 6 and Table 4 show differences between left infra-Hisian (n = 10) and left supra-Hisian VAs (n = 6). Left infra-Hisian VAs had a higher rate of inferior lead R-wave discordance (80% [8 of 10] vs 33.3% [2 of 6]; *P =* 0.006) (Supplemental Figure 5), and larger R-wave amplitude (vs preceding sinus beat) in lead I (100% [10 of 10] vs 66.4% [4 of 6]; *P =* 0.04) and lead aVL (90% [9 of 10] vs 50% [3 of 6]; *P =* 0.016). In contrast, left supra-Hisian VAs exhibited a larger R-wave amplitude (vs preceding sinus beat) in lead III (83.3% [5 of 6] vs 30% [3 of 10]; *P =* 0.042) (Supplemental Figure 4).

### Ablation outcomes of para-Hisian VAs

**Figure 7:**
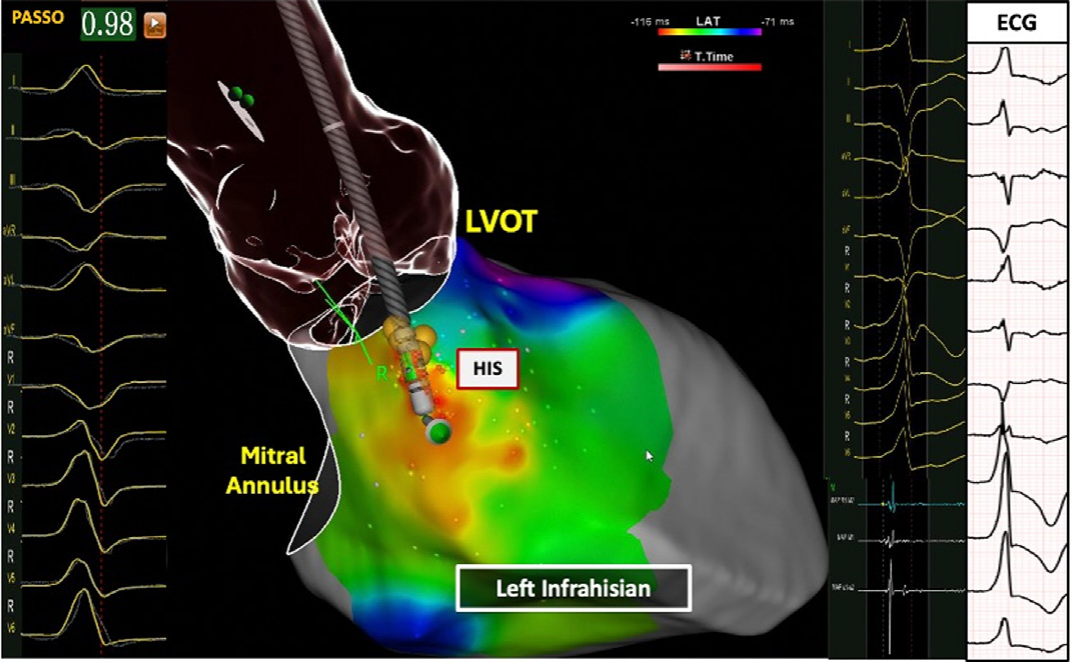
The Electroanatomical Features of Left Infra-Hisian Ventricular Arrhythmias This figure illustrates the electroanatomical location of left infra-Hisian ventricular arrhythmias, highlighting corresponding electrograms, ECG, and best pace match. The ECG, recorded at the same speed as the electrogram, shows a 98% pace match at the ablation site, with early signals marked in red. A straight catheter orientation was employed to counteract patient breathing movements. Left infra-Hisian ventricular arrhythmias exhibited a higher rate of inferior lead R-wave discordance (80% vs 33.3%; *P =* 0.006) and larger R-wave amplitudes in lead I (100% vs 66.4%; *P =* 0.04) and lead aVL (90% vs 50%; *P =* 0.016). Abbreviations as in Figure 6.

Figure 4, Figure 5, Figure 6, Figure 7 illustrate the electroanatomical characteristics, best pace match, and EGMs at the site of ablations for PH-VAs. Of the 31 patients treated, 22 (70.9%) received the STSF catheter, and 9 (29.1%) received the BW Q Dot catheter. The BW VIZIGO bidirectional sheath was used in 24 patients (77.4%) and the Agilis NxT Steerable sheath (Abbott Cardiovascular) was used in 7 patients (22.6%). Activation mapping showed the earliest activation occurred at a mean of 31 ± 6.7 ms pre-QRS interval. The mean pace match accuracy was 98.1% ± 1.1% and each patient received an average of 11.3 ± 4.3 lesions. RF energy delivery was delivered with an average output of 41.2 ± 2.4 W, with the target site located 9.5 ± 1.1 mm from the His Bundle. Immediate success was achieved in 87.1% (27 of 31) patients.

### Outcomes

**Figure 8:**
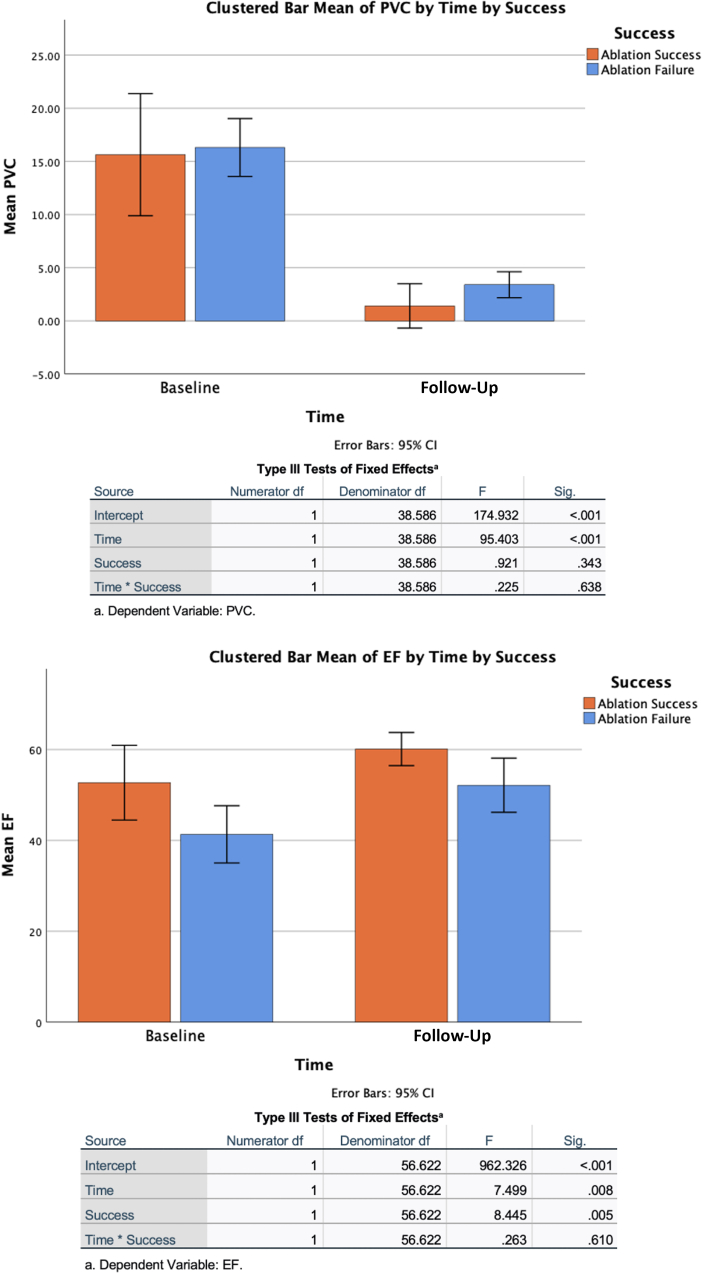
Left Ventricular EF and PVC Outcomes of the Different Para-Hisian Ventricular Arrhythmias This figure represents outcomes based on the mixed-effects model evaluating changes in left ventricular ejection fraction (EF) and premature ventricular contraction (PVC) burden in patients with Para-Hisian ventricular arrhythmias. Significant effects of time were observed for both left ventricular EF *(P =* 0.008) and PVC burden (*P <* 0.001). Group effects (ablation success vs failure) were significant for left ventricular EF *(P =* 0.005) but not for PVC burden *(P =* 0.343). No significant time-group interaction was found for either left ventricular EF *(P =* 0.610) or PVC burden *(P =* 0.638), suggesting similar changes across both groups over time.

**Figure 9:**
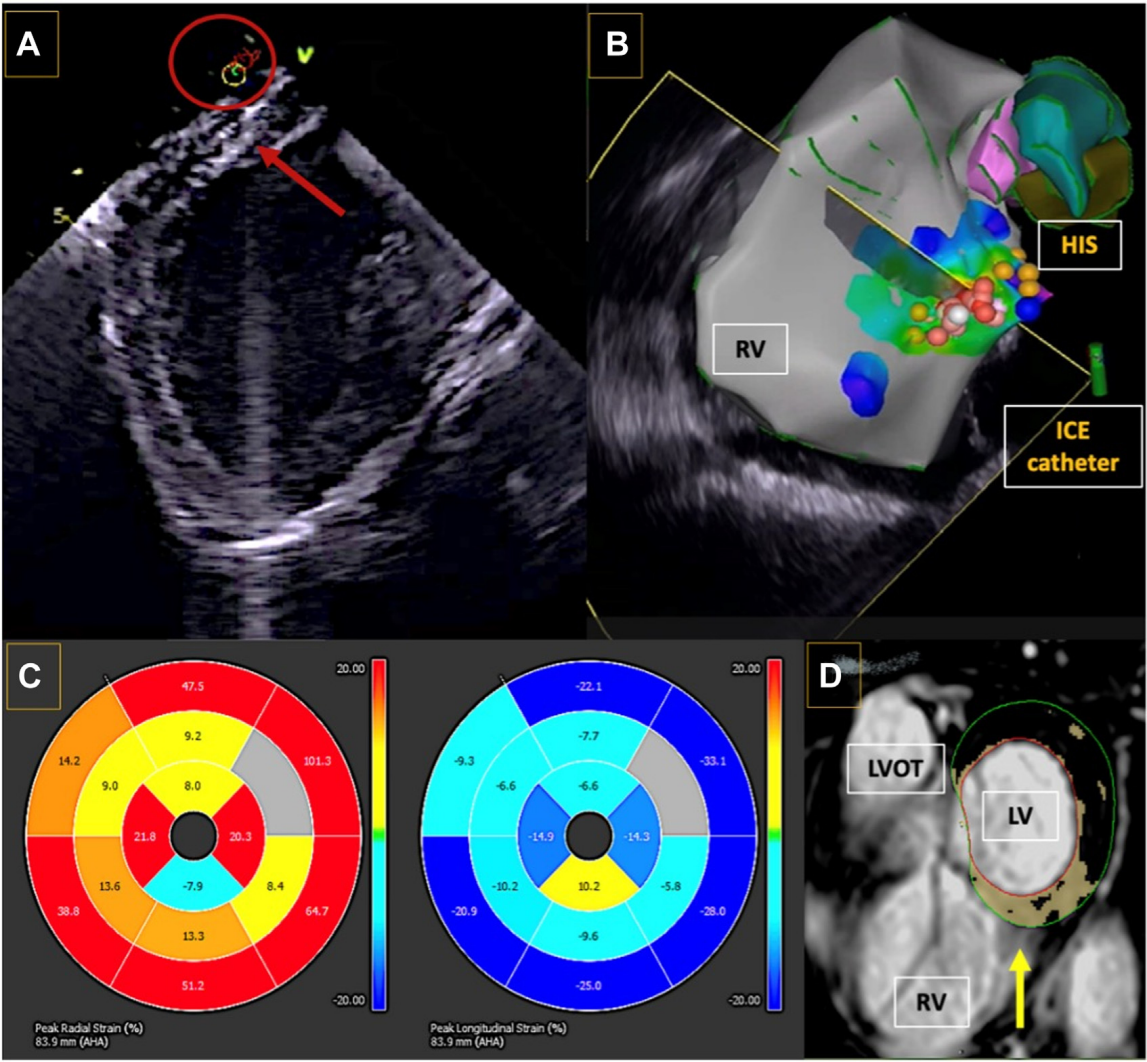
Role of Cardiac Magnetic Resonance Imaging and ICE in Guiding Ablations This figure illustrates the integration of intracardiac echocardiography (ICE) and cardiac magnetic resonance imaging in optimizing ablation strategies. (A) An ICE image highlighting the catheter's position and basal septal scar. (B) The ICE catheter fan at the ablation site. (C) Impaired radial and longitudinal strain on cardiac magnetic resonance imaging over the mid and basal septum, and (D) near transmural late gadolinium enhancement in the basal septum (yellow arrow), indicating significant myocardial fibrosis. LV = left ventricle, LVOT = left ventricular outflow tract; RV = right ventricle.

The median follow-up times were 15 months (Q1-Q3: 14-21 months) for left infra-Hisian VAs, 16 months (Q1-Q3: 14-20 months) for left supra-Hisian VAs, and 14 months (Q1-Q3: 14-16 months) for right infra-Hisian and right supra-Hisian VAs (Q1-Q/3: 14-15 months). During this period, changes in LVEF varied among the groups. The left supra-Hisian group showed a median improvement of 42.6% (Q1-Q3: 14.29%-78.13%), while the left infra-Hisian group had a median improvement of 14.64% (Q1-Q3: 0.00%-36.36%). The right supra-Hisian group had a median improvement of 16.67% (Q1-Q3: 3.33%-22.73%), while the right infra-Hisian group had a median improvement of 19.18% (Q1-Q3: 15.05%-33.74%). Regarding PVC burden, all groups saw reductions. The right supra-Hisian group had a reduction of 92.86% (Q1-Q3: 78.11%-99.29%), while the right infra-Hisian group had a reduction of 78.01% (Q1-Q3: 63.61%-92.21%). The left supra-Hisian group showed a reduction of 79.02% (Q1-Q3: 68.49%-98.93%), while the left infra-Hisian group had a reduction of 84.5% (Q1-Q3: 69.41%-97.69%). Figure 8 highlights the mixed-effects model analyzing changes in LVEF and PVC burden. At final follow-up, 16.1% (5 of 31) patients had devices implanted for conduction disease, ventricular tachycardia or heart failure. Post CA, only 1 patient developed heart block, and 16.1% (5 of 31) (Figure 9) patients had PR prolongation. Outcome measures for PH VAs are detailed in Table 5.

**Table 5:** Outcomes of the Different Para-Hisian Ventricular Arrhythmias

	Left Supra-Hisian	Left Infra-Hisian	*P* Value	Right Supra-Hisian	Right Infra-Hisian	*P* Value
Baseline EF	33.8 ± 11	51 ± 17.5	0.040	45.7 ± 12.8	45.2 ± 7.8	0.920
EF at 1-y follow-up, %	55 (45-57)	62 (55-67)		55 (47-60)	56.5 (55-60)	
Improvement in EF, %	42.6 (14.3-78.1)	14.6 (0.0-36.4)		16.7 (3.3-22.7)	19.2 (15.1- 33.7)	
Baseline PVC burden, %	18.4 ± 6.3	14.3 ± 11.4	0.435	13.7 ± 3.8	20.5 ± 3.2	0.002
PVC burden at 1-y follow-up, %	2.6 (0.19-7.30)	1 (0.10-3.1)		0.5 (0.10-3.70)	4.3 (1.65-6.50)	
Improvement in PVC burden), %	79 (68.5-98.9)	84.5 (69.4-97.7)		92.8 (78.1-99.3)	78 (63.6-92.2)	
Single procedure—drug-free outcome	1 (16.6)	2 (20)	0.870	3 (42.8)	1 (12.5)	0.630
Multiple procedures—drug-free outcome	0 (0)	1 (10)		0 (0)	2 (25)	
Single/multiple procedures + drug therapy	6 (100)	6 (60)	0.091	4 (57.1)	5 (62.5)	0.870
Device therapy before procedure	1 (16.6)	3 (30)	0.826	1 (14.2)	0 (0)	
Incidence of heart block	0 (0)	1 (10)		0 (0)	0 (0)	
Need for redo procedure	1 (16.6)	0 (0)		1 (14.3)	2 (25)	
Loss to follow-up	0 (0)	1 (10)		0 (0)	0 (0)	

Values are mean ± SD, median (Q1-Q3), or n (%).

EF = ejection fraction; PVC = premature ventricular complex.

### Quality of life improvement

**Figure 10:**
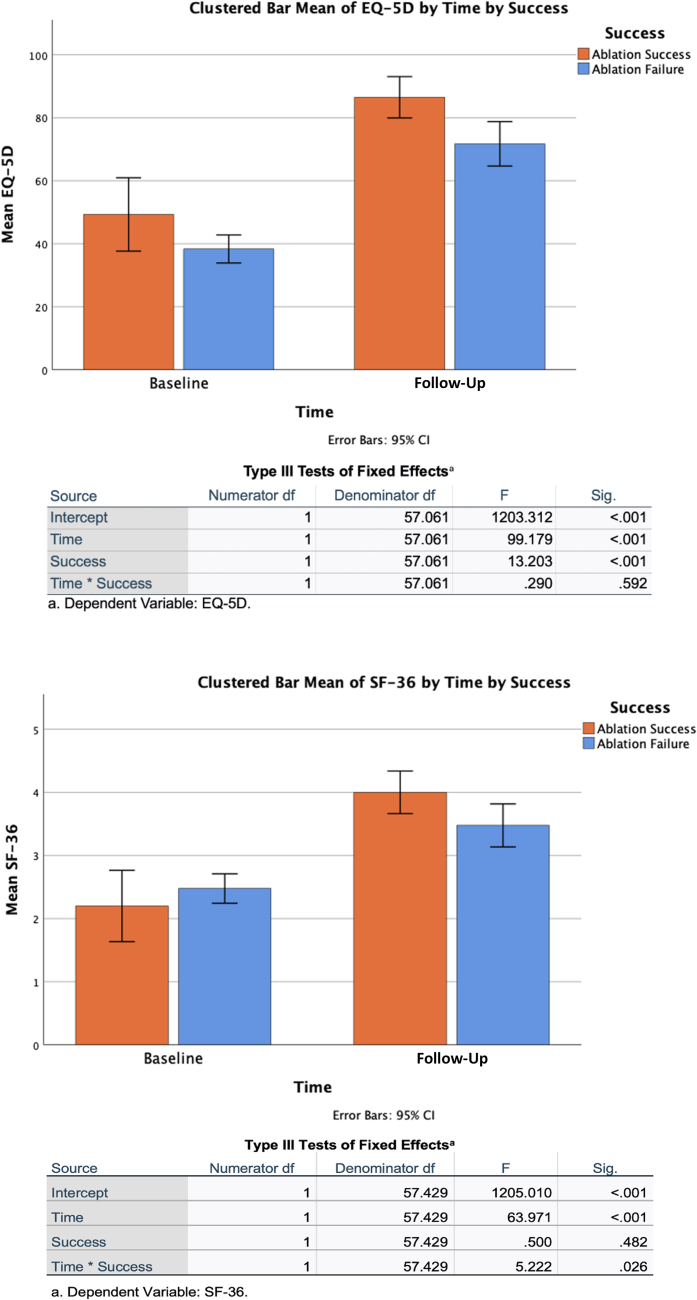
Quality-of-Life Outcomes of Para-Hisian Ventricular Arrhythmia Ablation This figure highlights the quality-of-life outcomes based on the mixed-effects model between patients with successful ablation and those with ablation failure. Quality-of-life parameters were evaluated using Short Form Health Survey 36 (SF-36) and EuroQol (EQ-5D) scores, revealing a significant interaction for SF-36 over time *(P =* 0.026), indicating improved scores in the successful ablation group. In contrast, while EQ-5D demonstrated significant main effects for time and group, the interaction was not significant *(P =* 0.592), suggesting no notable difference in EQ-5D changes between groups. These findings highlight the importance of successful ablation in enhancing patient quality of life.

Figure 10 highlights the mixed-effects model analyzing the QOL outcomes. QOL parameters were compared between patients who underwent successful ablation (single/multiple procedures without drug therapy) and those with ablation failure (single/multiple procedures with continued drug therapy). For SF-36, the mixed-effects model revealed a statistically significant interaction between time and group (ablation success vs failure) with a *P* value of 0.026. This indicates that changes in SF-36 scores between the 2 groups were significant over time. For EQ-5D, while there were significant main effects of time and group, the interaction between time and group was not statistically significant *(P =* 0.592), suggesting no significant difference in the changes between groups over time.

### Complications

In our cohort of patients, we had 2 patients with puncture site hematoma, but no significant vascular damage, stroke, or valvular insufficiency occurred. Additionally, 2 patients developed RBBB, and 1 patient with an existing permanent pacemaker (PPM) developed heart block intra-procedure.

## Discussion

The key findings of our study as shown in the Central Illustration include the following:

1. **PH vs Septal RVOT VAs:** PH VAs demonstrated the following: 1) narrower QRS complexes; 2) consistent presence of an R-wave in lead aVL; 3) inferior lead discordance; and 4) Early R-wave transition relative to the preceding sinus beat.
2. **Left vs Right PH-VAs**: Left PH VAs demonstrated a) earlier R-wave transition in lead V_1_/V_2_ and b) larger R-wave (vs preceding sinus beat) in lead V_2_. Right PH VAs demonstrated a larger S-wave (vs preceding sinus beat) in leads V_1_ and aVR.
3. **Left supra-Hisian vs Left infra-Hisian VAs**: Left supra-Hisian PH VAs demonstrated a larger R-wave (vs preceding sinus beat) in lead III. Left infra-Hisian VAs demonstrated a) greater inferior lead discordance and b) a larger R-wave (vs preceding sinus beat) in leads I and aVL.
4. **Right supra-Hisian vs Right infra-Hisian VAs**: Right supra-Hisian PH VAs demonstrated a larger S-wave (vs preceding sinus beat) in lead V_1_. Right infra-Hisian VAs demonstrated a larger S-wave (vs preceding sinus beat) in lead aVR.

**Central Illustration:**
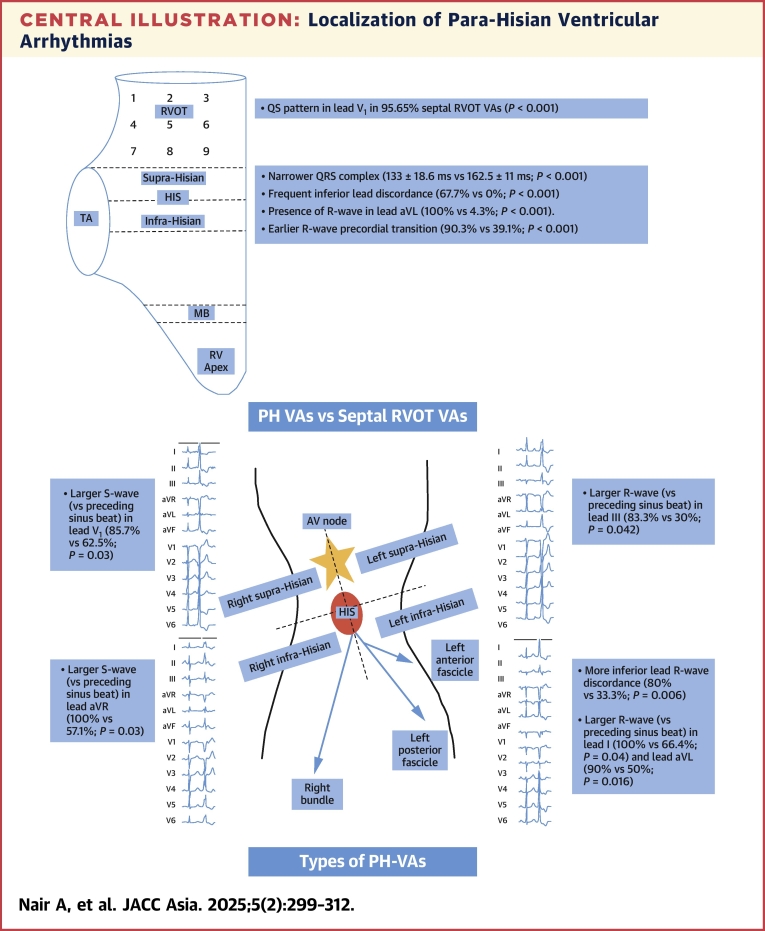
Localization of Para-Hisian Ventricular Arrhythmias This diagram illustrates the anatomical localization of Para-Hisian ventricular arrhythmias (PH) ventricular arrhythmias (VAs) and their relationship with septal right ventricular outflow tract (RVOT) VAs. Comparison between PH and septal RVOT VAs reveals that PH-VAs exhibit narrower QRS complexes, consistent R waves in lead aVL, inferior lead discordance, and early R-wave transition. Left PH VAs show earlier R-wave transition and larger R waves in lead V_2_, while right PH VAs present larger S waves in leads V_1_ and aVR. This delineation aids in understanding arrhythmia mechanisms and refining ablation strategies.

These findings align with previous studies, offering further insights into the differences between right and left PH-VAs, as well as supra- and infra-Hisian VAs. Enriquez et al^3^ identified distinctive ECG features in PH-VAs, including left bundle branch block pattern with a QS pattern in lead V_1_, early precordial transition (leads V_2_-V_3_), and narrow QRS duration. Ban et al^5^ described ECG features of VAs originating from the RV PH region and the posterior RVOT region, revealing an R-wave in leads I and aVL, and a narrower QRS width. Yamauchi et al^6^ noted similar characteristics of VAs near the His bundle. Anatomically, the His bundle’s posterior, lower, and rightward position within the RVOT shifts the depolarization vector leftward, resulting in R waves in leads I, aVL, and II. The earlier engagement of the His-Purkinje network explains the narrower QRS duration compared with RVOT VAs.^7^, ^8^, ^9^, ^10^, ^11^, ^12^ Liang et al^13^ described ECG features of VAs from the basal inferior-septum LV region such as R>S in lead V_1_, absence of QS pattern in the inferior leads, and shorter QRS duration.

Our study enhances the understanding of PH-VAs and is the first to systematically compare ECG features of PH-VAs to septal RVOT VAs and explore anatomical and ECG distinctions between right and left PH-VAs and supra-/infra-Hisian VAs. The use of advanced imaging techniques, such as ICE and 3D mapping, underscores their practical value in understanding the conduction system^14^ and improving procedural outcomes. Our findings confirm that PH-VA ablations improve LV function, reduce VA burden, and enhance QOL with a low incidence of heart block and only 1 patient lost to follow-up because of interstate migration (we continue to receive data from his cardiologist, confirming no recurrence of VA). Our study provides robust evidence supporting the efficacy of PH-VA ablations and offers valuable insights for future treatment strategies.

### Study limitations

ECGs are unipolar recordings of cardiac electrical activity and can be taken in both sitting and supine positions. Lead placement relative to the heart's orientation significantly affects ECG patterns. Accurate lead placement is crucial. In our study, patients with a vertically oriented heart, confirmed by computed tomography, showed no discordance in the inferior leads.

## Conclusions

VAs often originate from the PH region and exhibit distinct ECG patterns. Effective treatment of these VAs depends on a deep understanding of specific ECG characteristics, use of advanced mapping technologies, and precise localization.

## Funding Support and Author Disclosures

This research did not receive any specific grant from funding agencies in the public, commercial, or not-for-profit sectors. Dr Pathak has served on the advisory board of Medtronic, Abbott, and Biosense Webster; Canberra Heart Rhythm Foundation/Australian National University has received on his behalf lecture and/or consulting fees/research funding from Medtronic, Abbott, Boston Scientific, Biotronik, and Biosense Webster. Dr Sanders has served on the advisory board of Medtronic, Abbott, Boston Scientific, CathRx, and PaceMate; and the University of Adelaide has received on his behalf lecture and/or consulting fees/research funding from Medtronic, Abbott, Microport, and Boston Scientific. All other authors have reported that they have no relationships relevant to the contents of this paper to disclose.
