# Supplementary material for: Electrocardiographic Characteristics and Ablation Outcomes Associated With Para-Hisian Ventricular Arrhythmias
Source: JACC Asia. 2025 Jan 14;5(2):299–312. doi: 10.1016/j.jacasi.2024.11.012 (PMC11840233; doi:10.1016/j.jacasi.2024.11.012)

**Electrocardiographic Characteristics and Ablation Outcomes Associated With Para-Hisian Ventricular Arrhythmias**

Anugrah Nair DM ^1,2^; Jenish P Shroff DM ^1,2^; Lukah Q. Tuan ^1,2^; Adriana Tokich BSc^1,2^; Deep Chandh Raja DM^1,2^, Abhinav Mehta B.Sc^1^; Walter P Abhayaratna MBBS, PhD^1^; Prashanthan Sanders MBBS, PhD,^3^; Francis E. Marchlinski, MD^4^; Kalyanam Shivkumar MD PhD^5^**;** Rajeev K Pathak MBBS, PhD ^1,2^

1. **Supplemental** **Figure 1: The electrocardiographic characteristics of para-hisian VAs in presence of myocardial scar..**   
   Figure highlights distinct ECG patterns in presence and absence of scar. Case A is PH-VA without scar and case B is PH-VA in presence of scar. Among the 16 patients with myocardial scar, 62.5%(10/16) patients had a QRS duration >150ms and all exhibited notching of the QRS complexes on their ECGs. Notching was present in multiple leads in 9 patients, whereas 7 patients showed notching in a single lead. **Abbreviations**; ECG: electrocardiogram; PH-VA: para-hisian ventricular arrhythmia; RVOT: right ventricular outflow tract.


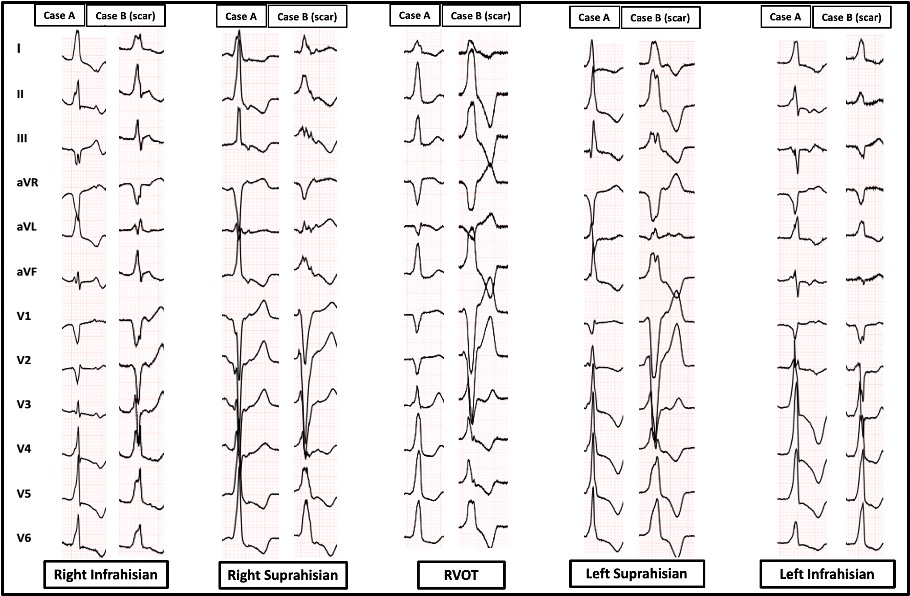


**Supplemental Figure 2:** **Electrocardiogram examples of right supra-hisian VAs.**

Figure highlights the ECGs of all the right supra-hisian VAs. Right supra hisian PH VAs demonstrated a larger S wave (vs preceding sinus beat) in lead V1. **Abbreviations**; ECG: electrocardiogram; VA: ventricular arrhythmia.


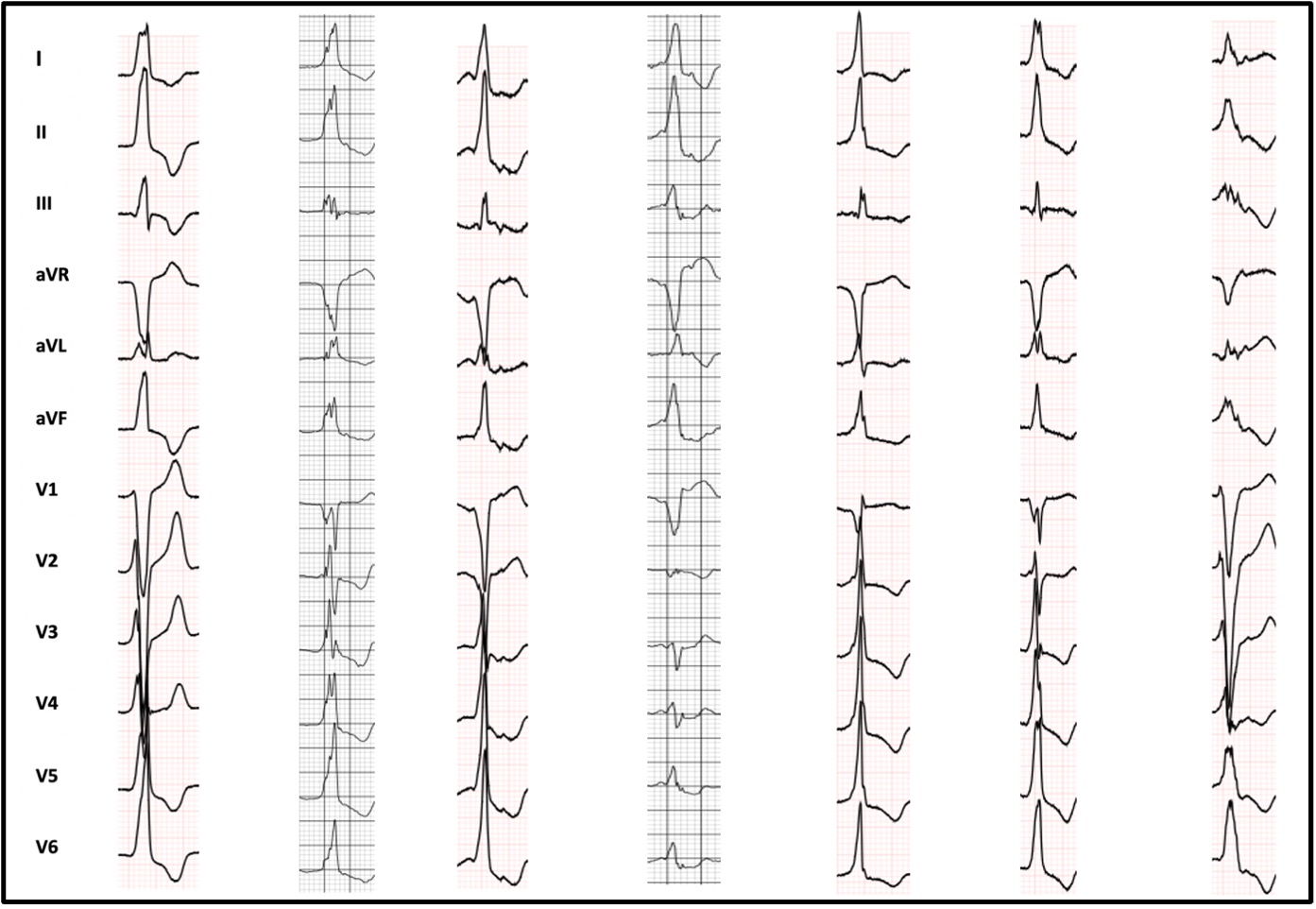


**Supplemental Figure 3:** **Electrocardiogram examples of right infra-hisian VAs.**

Figure highlights the ECGs of all the right infra-hisian VAs. Right infra hisian VAs demonstrated a larger S wave (vs preceding sinus beat) in leads aVR. **Abbreviations;** ECG: electrocardiogram; VA: ventricular arrhythmia.


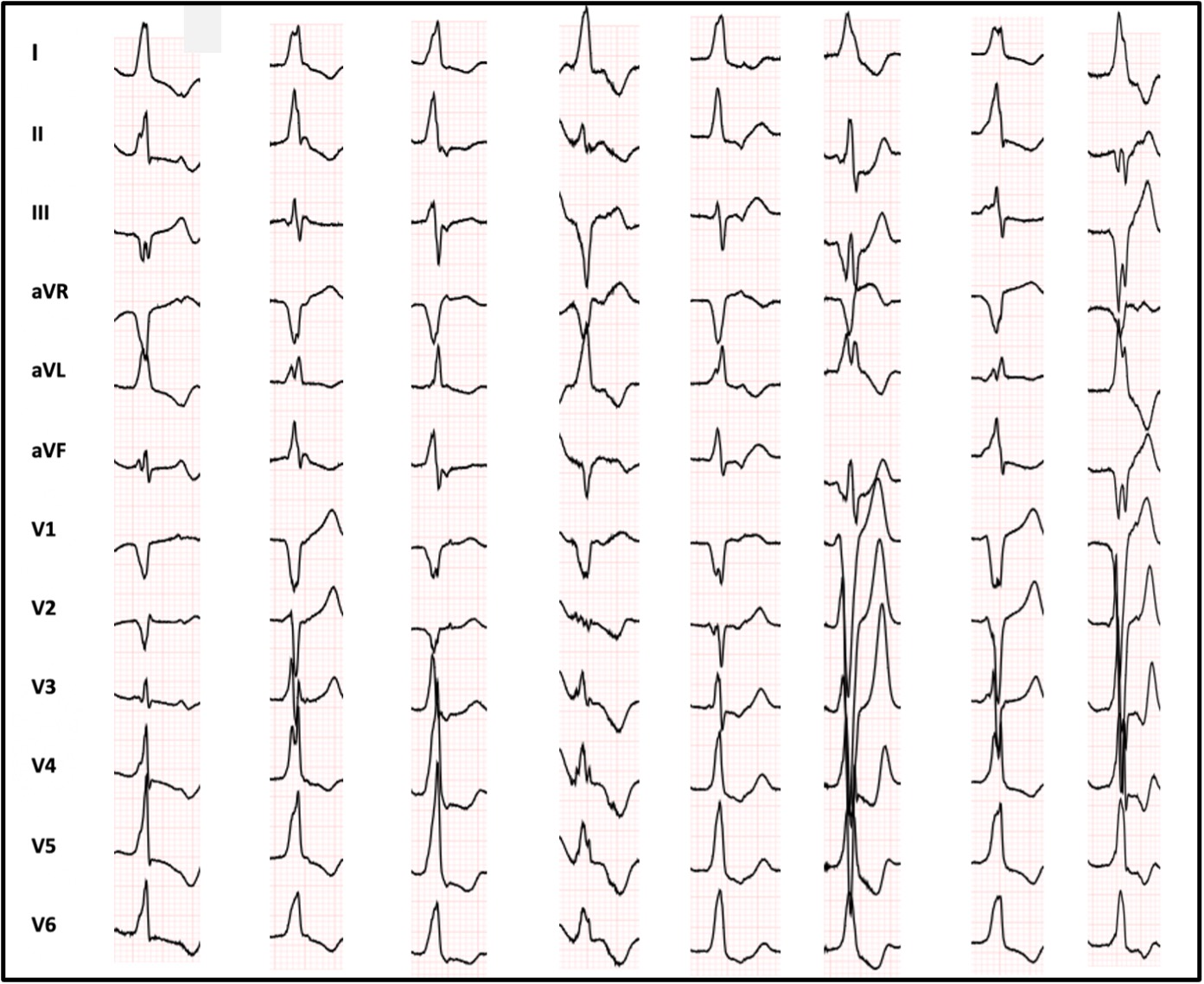


**Supplemental Figure 4:** **Electrocardiogram examples of left supra-hisian VAs.**

Figure highlights the ECGs of all the left supra-hisian VAs. Left supra hisian PH VAs demonstrated a larger R wave (vs preceding sinus beat) in lead III. **Abbreviations**; ECG: electrocardiogram; VA: ventricular arrhythmia.


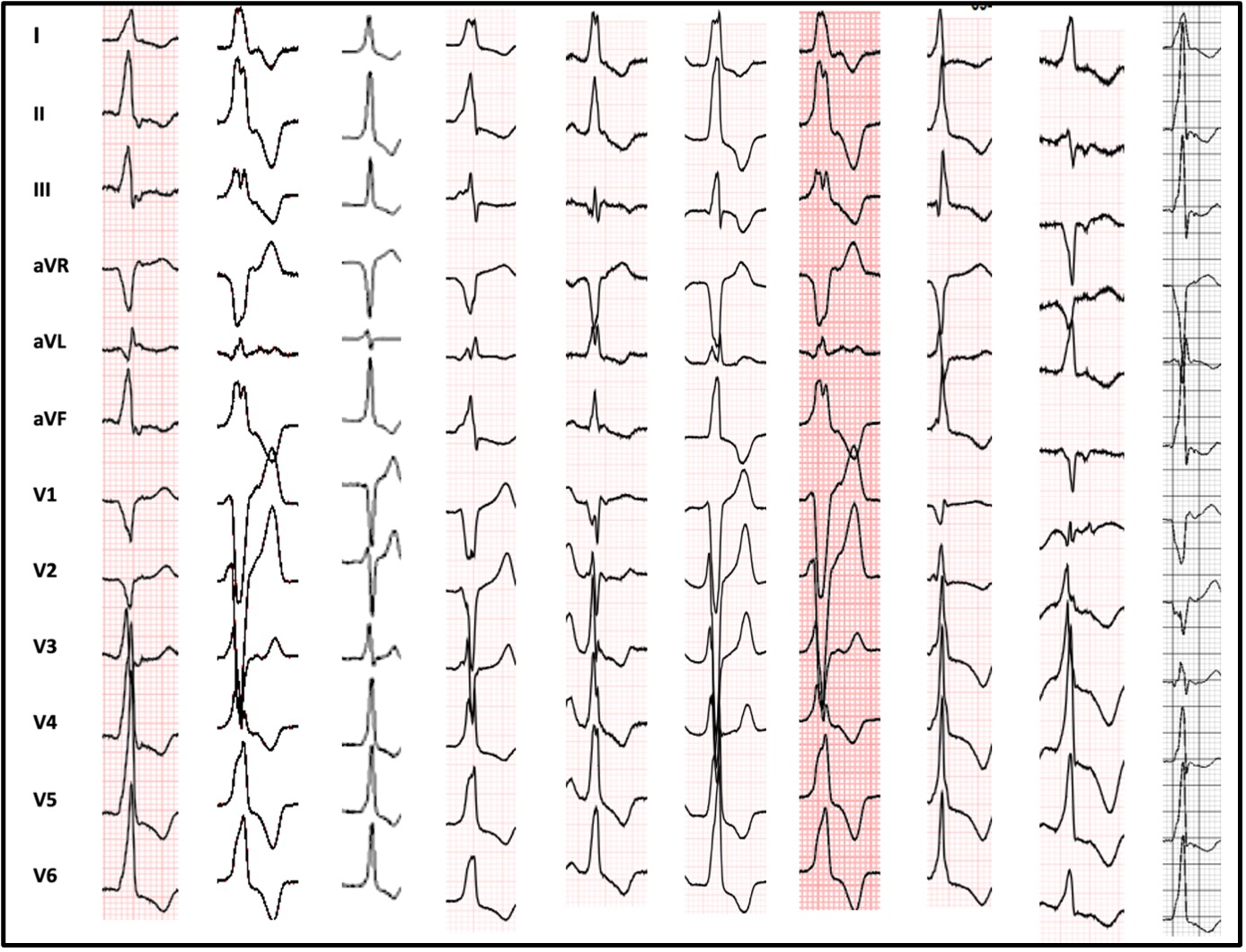


**Supplemental Figure 5:** **Electrocardiogram examples of left infra-hisian VAs.**

Figure highlights the ECGs of all the left infra-hisian VAs. Left infra hisian VAs demonstrated a) greater inferior lead discordance and b) a larger R wave (vs preceding sinus beat) in leads I and aVL. **Abbreviations**; ECG: electrocardiogram; VA: ventricular arrhythmia.


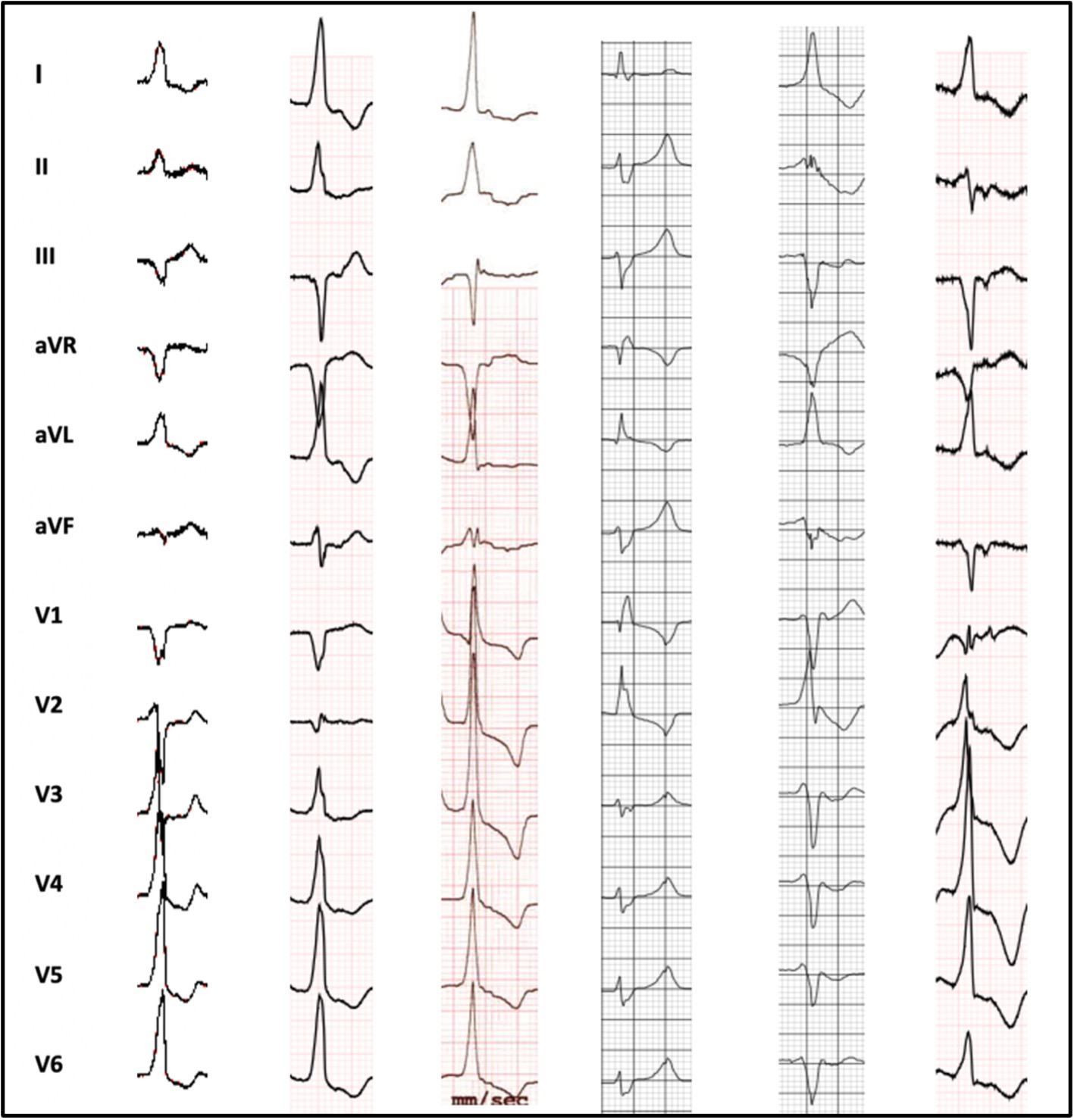

Supplement: Supplemental Figures 1-5 [file mmc1.docx]
